# KLRG1 and CD103 Expressions Define Distinct Intestinal Tissue-Resident Memory CD8 T Cell Subsets Modulated in Crohn's Disease

**DOI:** 10.3389/fimmu.2020.00896

**Published:** 2020-05-12

**Authors:** Hugo Bottois, Marjolaine Ngollo, Nassim Hammoudi, Tristan Courau, Julie Bonnereau, Victor Chardiny, Céline Grand, Brice Gergaud, Matthieu Allez, Lionel Le Bourhis

**Affiliations:** ^1^Université de Paris, INSERM U1160, EMiLy, Institut de Recherche Saint-Louis, Paris, France; ^2^Gastroenterology Department, Hopital Saint Louis, AP-HP, Paris, France

**Keywords:** T cells, IBD – inflammatory bowel diseases, Crohn's disease, TRM cells, CD103, KLRG1

## Abstract

Intestinal tissue-resident memory CD8 T cells (Trm) are non-recirculating effector cells ideally positioned to detect and react to microbial infections in the gut mucosa. There is an emerging understanding of Trm cell differentiation and functions, but their implication in inflammatory bowel diseases, such as Crohn's disease (CD), is still unknown. Here, we describe CD8 cells in the human intestine expressing KLRG1 or CD103, two receptors of E-cadherin. While CD103 CD8 T cells are present in high numbers in the mucosa of CD patients and controls, KLRG1 CD8 T cells are increased in inflammatory conditions. Mucosal CD103 CD8 T cells are more responsive to TCR restimulation, but KLRG1 CD8 T cells show increased cytotoxic and proliferative potential. CD103 CD8 T cells originate mostly from KLRG1 negative cells after TCR triggering and TGFβ stimulation. Interestingly, mucosal CD103 CD8 T cells from CD patients display major changes in their transcriptomic landscape compared to controls. They express Th17 related genes including CCL20, IL22, and IL26, which could contribute to the pathogenesis of CD. Overall, these findings suggest that CD103 CD8 T cells in CD induce a tissue-wide alert increasing innate immune responses and recruitment of effector cells such as KLRG1 CD8 T cells.

## Introduction

T cells differentiate from naive precursors after recognition of their specific antigen, (1) generating central and effector memory T cells (Tcm, Tem) which are defined by different functions (2, 3). Recent studies describe a subset of memory T cell located in tissues, resident memory T cells (Trm) defined by their retention at mucosal sites such as the skin, lung and intestine (4, 5). Trm cells have distinct transcriptional and phenotypic signatures compared to blood memory T cells (6–8). Trm cell differentiation relies on the capacity of effector T cells to migrate into tissues and respond to local tissular cues, such as chemokines and adhesion molecules, produced constitutively or during infection and inflammation (9, 10).

In the intestine, the presence of the microbiota and foodborne antigens generate a tissular microenvironment that participates to the presence of T cells with specific features. Indeed, intestinal specific signals from the microbiota and inflammatory pathways, such as TGFβ or IL-15, have been suggested to regulate the phenotype and functions of Trm cells in the intestine (11–13). Hence, T cells are rewired *in situ* following their recruitment in the gut and acquire new functions fitting to maintain intestinal homeostasis, respond to potential infections while preventing unnecessary immune responses to harmless protein antigens and commensal bacteria (9, 13–15).

The C-Type lectin CD69 and the integrin CD103 have been used as surrogate markers to identify Trm cells. CD69 is an early activation marker of T cells that promotes retention in the tissue (9, 16). CD103 is formed by the dimerization of the integrins αE and β7, and interacts with E-cadherin expressed by intestinal epithelial cells (IEC). However, while almost all CD8 Trm cells express CD69, a significant number of them lack CD103 expression, which suggests heterogeneity in CD8 Trm cell populations (5, 6, 17). Indeed, CD8 T cells activated through TCR stimulation and inflammatory signals can also express KLRG1, which can compete with CD103 for its interaction with E-cadherin in the mucosa (18, 19). Interestingly, studies in mice show that CD103 expressing CD8 Trm cells derived from KLRG1 negative precursors (9, 14, 20). Recently, CD8 T cells in the human intestine have been shown to recapitulate these features in a model of intestinal transplantation. In this model, tracking of immune cells from the intestine donor and from the recipient allowed to determine that duodenal CD103 CD8 Trm cells are long-lived cells present in both the lamina propria and intraepithelial compartments, while KLRG1 CD8 T cells are distinct in their phenotype and are replenished from the recipient peripheral blood (21). These observations suggest that several subsets of CD8 Trm cells could be present and have different functions in the human intestinal mucosa.

CD8 Trm cells are thought to play an important role in the physiopathology of tissular chronic diseases (22, 23) such as psoriasis (24) in the skin or celiac disease in the intestine (25). We recently showed that intestinal CD8 T cells clonal expansions are associated with post-operative recurrence in Crohn's Disease (CD) patients (26). However, the differentiation pathways and functions of CD8 Trm cells in the human intestinal mucosa and their role in the physiopathology of inflammatory bowel diseases (IBD) such as CD remain poorly understood.

In this study, we show that expression of CD103 contrasted with KLRG1 defining two populations of effector CD8 T cells. CD103 positive CD8 T cells mostly derived from KLRG1 negative CD8 T cells and were more responsive to TCR triggering, while KLRG1 CD8 cells expressed higher levels of granzyme B (GZM B). In the intestine, KLRG1 CD8 cells were increased in CD patients compared to controls. Comparative gene expression studies between intestinal CD8 subsets showed major changes in the transcriptomic landscape of CD103 positive cells compared to their CD103 negative counterparts. CD103 CD8 Trm cells from CD patients expressed more Th17 related cytokines as well as GZM K and NKG2A than in healthy individuals, which could participate to the pathogenesis of CD. Hence, CD103 and KLRG1 CD8 Trm cells could define two distinct subsets with different functions in the human intestinal mucosa.

## Results

### CD103 and KLRG1 Expressions Define Two Population of Effector CD8 T Cells

**Figure 1:**
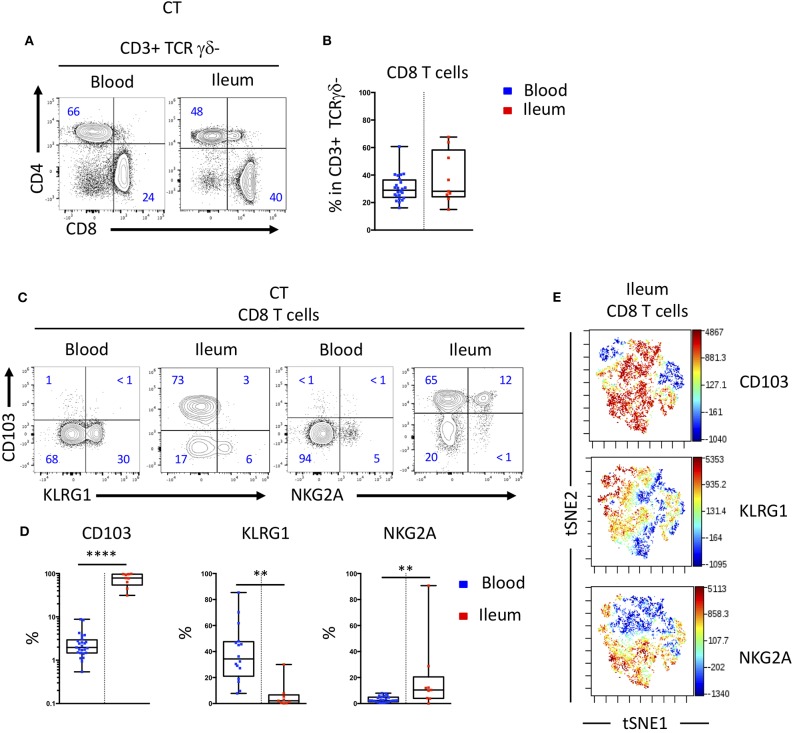
CD103 and KLRG1 expressions on CD8 T cells are mutually exclusive in blood and intestinal mucosa. **(A)** Representative staining and **(B)** proportions of CD4 and CD8 T cell subsets in blood and ileal mucosa of control individual (CT) (*n* = 25 and *n* = 10 for blood and ileum, respectively; boxes represent median and range). **(C)** Representative staining and **(D)** quantification of CD103, KLRG1 and NKG2A expression on CD8 T cells in blood and ileal mucosa of control individuals (CT) (*n* = 25 and *n* = 10 for blood and ileum, respectively; boxes represent median and range; unpaired *t*-test: **p < 0.001, ****p < 0.00001). **(E)** tSNE analysis of CD103, KLRG1 and NKG2A expression on ileal mucosal CD8 T cells.

We studied the proportion of human T cell subsets in the blood and the intestinal mucosa and observed similar proportion of CD8 T cells in these two compartments (Figures 1A,B). We next assessed the expression of CD103 and KLRG1, two receptors of E-cadherin expressed by intestinal epithelial cells, on peripheral and mucosal CD8 T cells and saw an expected increased expression of CD103 on mucosal CD8 T cells compared to peripheral CD8 T cells. In contrast, KLRG1 was more expressed by blood cells compared to mucosal CD8 T cells (Figures 1C,D). In the blood, KLRG1 expression was mostly restricted to CD8 Tem cells compared to Tcm and mucosal T cells (Supplementary Figures 1A,B). Blood KLRG1 positive CD8 T cells do not express the tissue retention marker CD69 (Supplementary Figures 2A,B) compared to their mucosal counterparts suggesting that they undergo further differentiation within the tissue.

Interestingly, expressions of CD103 and KLRG1 on CD8 T cells were mostly exclusive in both blood and mucosa (Figures 1C,E). Most CD8 T cells in the intestine expressed CD103 and high levels of CD69, while a smaller population of cells expressed CD69 but were negative for CD103 and could expressed KLRG1 (Supplementary Figures 2C,D). The inhibitory receptor NKG2A was co-expressed mainly by CD103 positive CD8 T cells in the mucosa (Figures 1C–E). Taken together these results suggest that expression of CD103 and KLRG1 markers could define functionally different subsets of CD8 T cells in the intestine.

### TGFβ and TCR Triggering Induces CD103 Expression on T Cells, but Decrease KLRG1 Expression

**Figure 2:**
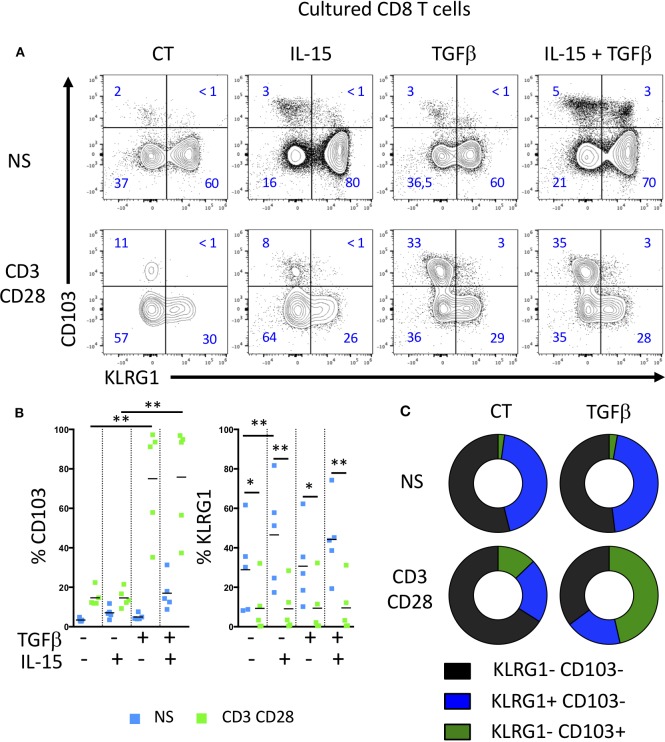
TGFβ and TCR triggering highly induces CD103 expression on T cells, but decrease KLRG1 expression. **(A)** Representative expression of CD103 and KLRG1 on CD8 T cells following IL-15 and TGFβ incubation and TCR stimulation during 7 days. **(B)** Quantification and **(C)** proportion of CD103 and KLRG1 expression on CD8 T cells (dots and bars represent 5 independent experiments with mean; ANOVA with Bonferoni post-test:*p < 0.05, **p < 0.001).

We next analyzed the acquisition of CD103 and KLRG1 by CD8 T cells upon TCR triggering and in the presence of soluble mediators locally produced in the intestinal environment. We thus incubated peripheral CD8 T cells from healthy donors with TGFβ and/or IL-15 to simulate healthy or inflammatory tissular conditions, with or without TCR triggering to mimic the presence or absence of a specific antigen. TGFβ alone or in combination with IL-15 failed to increase significantly the expression of CD103, while stimulation of CD8 T cells with TGFβ and TCR triggering strongly induced CD103 expression (Figures 2A–C). In contrast, KLRG1 expression was decreased on CD8 T cells upon TCR stimulation (Figures 2A–C). These data suggest that TGFβ and TCR signals differentially regulate the acquisition of CD103 and KLRG1 on CD8 T cells.

### CD103 CD8 T Cells Originate From KLRG1 Negative CD8 T Cells After TGFβ Exposure and TCR Triggering

**Figure 3:**
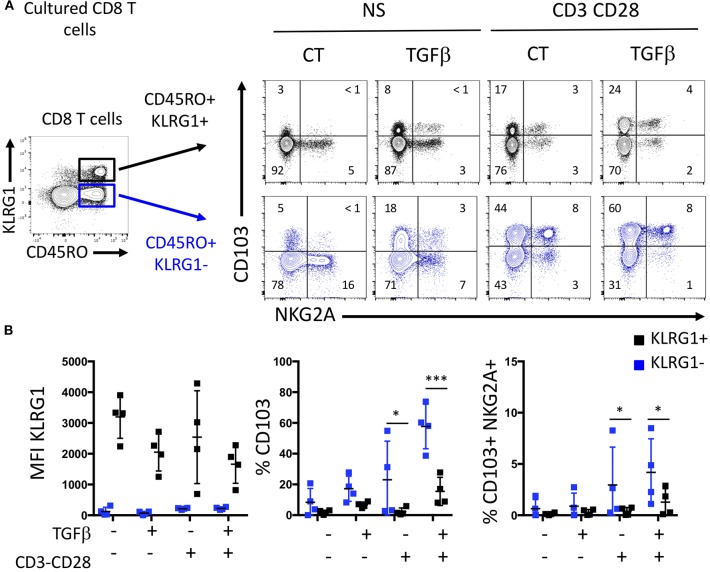
KLRG1-CD8 T cells acquire CD103 following TGFβ exposure and TCR triggering. **(A)** Representative expression of C0103 and NKG2A on sorted memory KLRG1- and KLRG1+ after incubation with or without TGFβ and TCR triggering. **(B)** Quantification of C0103, KLRG1, and C0103+NKG2A+ expression on C08 T cells (dots and bars represent 4 independent experiments with mean and SEM; ANOVA with Bonferoni post-test: *p < 0.05, ***p < 0.0001).

Based on mice studies (8, 14) showing that CD103 T cells stem from a blood memory precursor lacking KLRG1, we tested the ability of human memory cell subsets to acquire CD103 under TGFβ exposure and TCR stimulation. Blood memory CD45RO CD8 T cells were sorted according to KLRG1 expression. Memory KLRG1 negative CD8 T cells strongly acquired CD103 upon TCR and TGFβ triggering, while sorted KLRG1 positive cells did not (Figures 3A,B). We also see that some CD103 cells can be generated from the KLRG1 positive cells in our experiment, suggesting that the plasticity observed in the mouse model could exist in human also. However, effector KLRG1 negative cells generated three times more CD103 cells (Figures 3A,B), indicated that they are more equipped to do so. The coexpresssion of CD103 and NKG2A on CD8 T cells observed in the intestinal mucosa was achieved predominantly with KLRG1 negative memory CD8 T cells (Figures 3A,B). This suggests that the phenotype and functions of mucosal CD103 and KLRG1 Trm cells could result from distinct differentiation programs from memory CD8 T cells.

### KLRG1 Expressing CD8 T Cells Is Associated With Inflammation in CD

**Figure 4:**
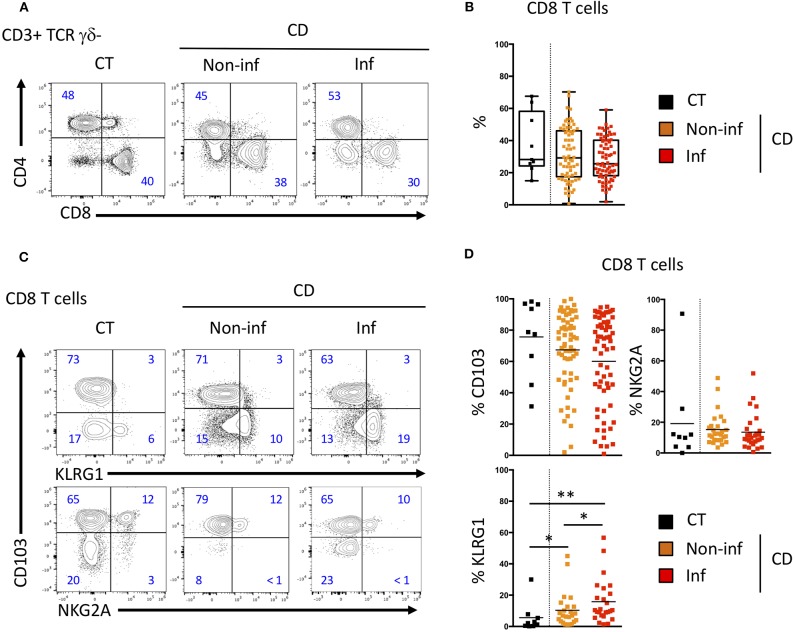
CD103 and KLRG1 CD8 Trm cell subsets in ileal Crohn's disease. **(A)** Representative CD4 and CD8 T cells staining and **(B)** quantification in the ileal mucosa of control individuals (CT; *n* = 10), CD patients in macroscopically inflamed (lnf) and non-inflamed (non-inf) segments of the ileocecal resections (*n* = 69; boxes represent median and range; unpaired *t*-test: not significant). **(C)** Representative expression and **(D)** quantification of CD103 (*n* = 69), KLRG1 (*n* = 25), and NKG2A (*n* = 35) on CD8 T cells from ileal mucosa as in A (bars represent means; unpaired *t*-test between CT and CD; paired *t*-test between lnf and Non-lnf: *p < 0.05, **p < 0.001).

We then studied these CD8 T cell subsets in patients with Crohn's disease undergoing ileocecal resection surgery. We analyzed the phenotype and proportion of CD8 T cells in control ileum (CT), the inflamed mucosa (Inf) as well as the macroscopically non-inflamed ileal margins (Non-inf) of the surgical specimens. The proportion of CD8 T cells of CD patients was similar to what was observed in CT (Figures 4A,B).

We then compared the expression of CD103, KLRG1 and NKG2A on intestinal CD8 T cells from normal and inflammatory ileal mucosa (Figures 4C,D). CD103 and NKG2A expressing CD8 T cells were in similar proportion in mucosa from controls (CT) and inflamed or non-inflamed mucosa from CD patients (Figures 4C,D). CD103 expression was decreased in inflamed compared to non-inflamed ileal mucosa but this difference did not reach statistical significance. However, proportions of KLRG1 expressing CD8 T cells in the intestinal mucosa of CD patients was significantly increased compared to CT (Figures 4C,D) and, KLRG1 CD8 T cells were in greater proportion in inflamed mucosa of CD patients compared to non-inflamed ileal samples from the same patients (Figures 4C,D). Interestingly, blood KLRG1 CD8 T cells expressed the chemokine receptor, CCR6 (Supplementary Figures 1C,D), which enable their migration into the tissues. These results suggest that proportion of CD103 and KLRG1 expressing CD8 Trm cells are associated with the inflammatory status in the intestine.

### Differential Effector Functions of CD103 Positive and Negative Mucosal CD8 Trm Cells

**Figure 5:**
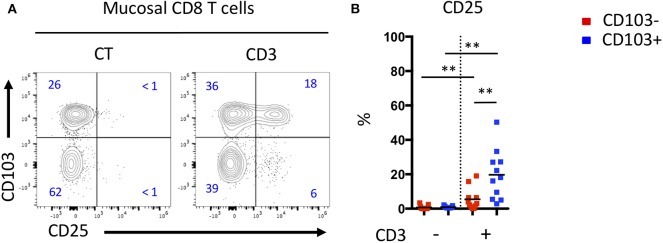
Mucosal CD103+ CD8 T cells from CD patient are more responsive to TCR triggering than CD103- CD8 T cells. **(A)** Representative expression of CD103 and CD25 activation marker on mucosal CD8 T cells from CD patients after TCR triggering. **(B)** Quantification of CD25 expression on CD103+ and CD103- mucosal CD8 T cells from CD patients after TCR triggering (*n* = 11, bars represent means, paired *t*-test, **p < 0.001).

Differences in the differentiation and proportion of these CD8 T cell subsets suggest that they may have diverse functions. We tested the responsiveness of mucosal CD8 T cells from CD patient by CD3 triggering and visualization of the activation marker, CD25. We noticed that for the same TCR stimulation, CD103 positive Trm cells express more CD25 than CD103 negative cells (Figures 5A,B). These results suggest that mucosal CD103 positive CD8 Trm cells were more responsive to TCR triggering than CD103 negative cells.

**Figure 6:**
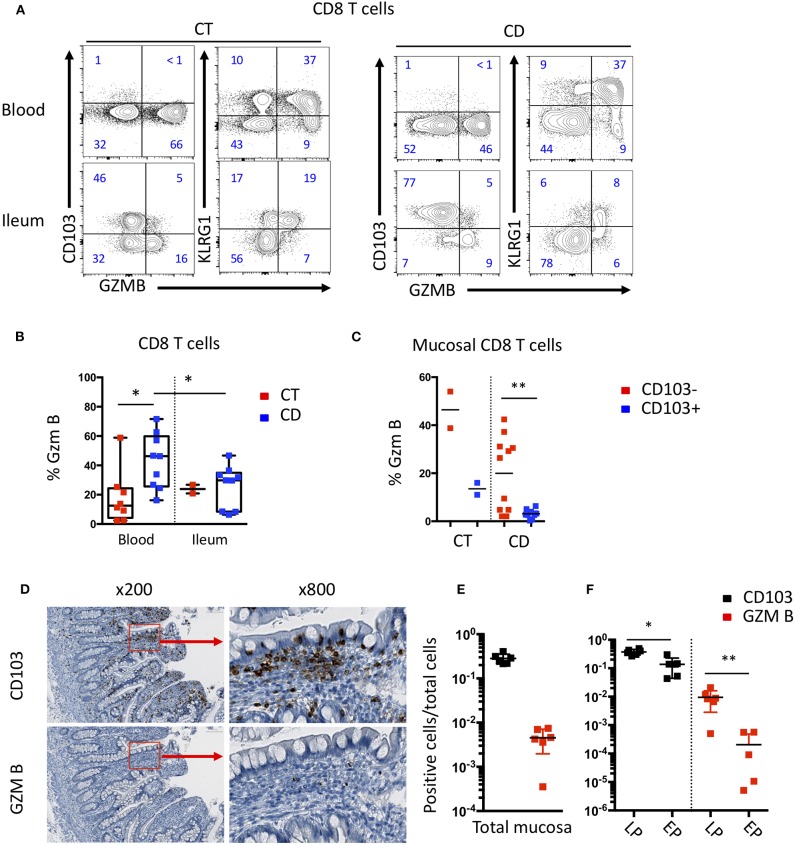
Differential effector functions of CD103 positive and negative mucosal CD8 T cells. **(A)** Representative expression of CD103, KLRG1 and GZMB on CD8 T cells in blood and ileal mucosa of control individuals (CT) and Crohn's disease (CD) patients. **(B)** Quantification of GZMB expression on CD8 T cells from blood and ileal mucosa (CT *n* = 2; CD *n* = 10; bars represent means; unpaired and paired *t*-test: *p < 0.05) **(C)** Quantification of GZMB expression on ileal CD103+ and CD103- CD8 T cell subsets (CT *n* = 2; CD *n* = 10; bars represent means; paired *t*-test: **p < 0.001). **(D)** Representative immunostaining of CD103 and GZMB on intestinal mucosa of CD patient. **(E)** Ratio of CD103 or GZM B positive cells on total mucosal cells (*n* = 6; bars represent means; paired *t*-test: not significant). **(F)** Ratio of CD103 or GZM B positive cells on cells in the Lamina propria (LP) or epithelium (EP) (*n* = 6; bars represent means; paired *t*-test: *p < 0.05, **p < 0.001).

To assess the effector potential of these CD8 T cell subsets in the blood and mucosa of CD patients, we analyzed the expression of effector molecules such as Granzyme B (GZM B) and the proliferative marker Ki67 by intracellular staining (Supplementary Figure 3A). A higher proportion of CD8 T cells from the blood of CD patients expressed GZM B compared to CT (Supplementary Figure 3B). In CD patients, CD8 Trm cells in the mucosa expressed higher levels of Ki67 than in the blood (Supplementary Figure 3B). Interestingly, KLRG1 CD8 T cells, in the mucosa and in peripheral blood, expressed higher levels of GZM B than CD103 positive CD8 Trm cells (Figures 6A,B). The expression of GZM B and Ki67 were significantly increased in CD103 negative cells in the mucosa compared to their CD103 positive counterparts (Figure 6B). These results suggest that KLRG1 positive CD8 T cells have higher proliferative and cytotoxic potential compared to CD103 positive cells in the mucosa.

We next analyzed the expression of CD103 and GZM B in the mucosa of CD patients by immunochemistry to determine the localization of these different CD8 T cell subsets. We observed that CD103 cells clearly outnumbered GZM B positive cells (Figures 6C,D). Cells expressing CD103 were mainly located in or near the epithelium, while cells expressing GZM B were mainly located in the lamina propria (Figures 6E,F). These results suggest that the different subsets of CD8 T cells have different location and potential cellular partners within the intestinal mucosa.

### Major Transcriptional Changes Between CD103- and CD103+ CD8 Trm Cells From CD Patient

**Figure 7:**
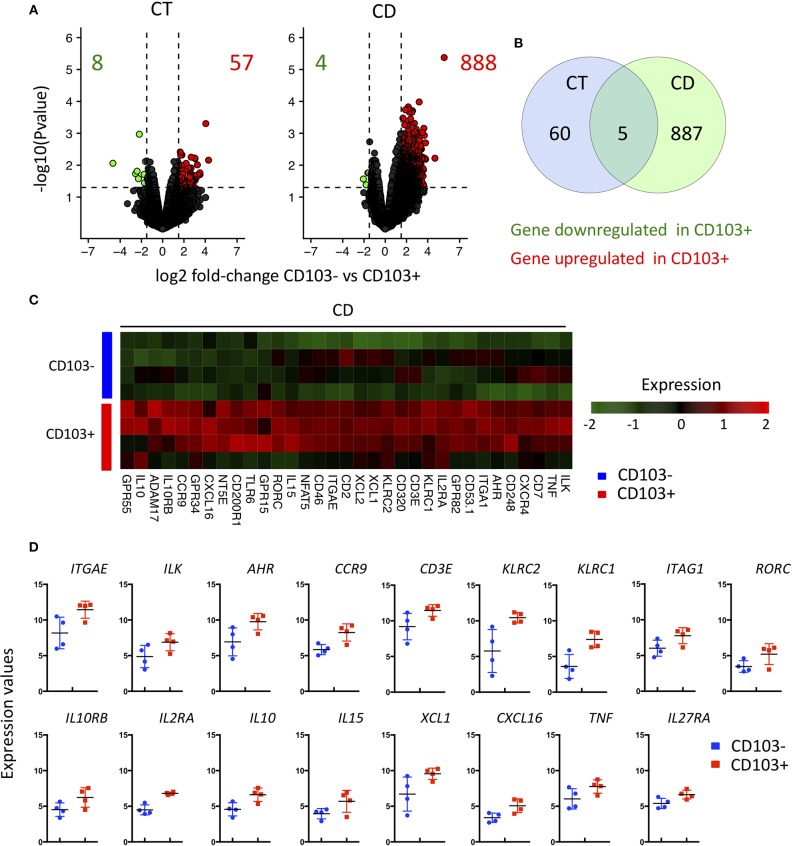
Major transcriptional change between CD103- and CD103+ CD8 Trm cells from CD patient. **(A)** volcanoplot showing differential expression (fold change >1.5 and *P* < 0.05) between CD103- and CD103+ T cells subsets from control tissue (CT; *n* = 3) and CD patients (CD; *n* = 4) as indicated. **(B)** Venn diagram showing number of gene differentially expressed between CD103- and CD103+ T cells subsets of CT and CD patients and overlapped gene. **(C)** Heatmap of genes differentially expressed (fold change >1.5 and *P* < 0.05) between CD103- and CD103+ mucosal T cells from CD patients. **(D)** Quantification and distribution of expression value of genes of interest (dots and bars represent 4 independent experiments with mean and SEM).

To further characterize the differences between CD8 T cells CD103 positive and negative (hence including all KLRG1 positive cells), we sorted these subsets from CT and CD patients and performed transcriptomic analyses. Several genes were differentially expressed when comparing CD103 positive and negative CD8 T cells from controls (Figure 7A). These differences were strikingly more important when we compared CD103 positive and negative CD8 T cells from CD patients, with almost 900 genes differentially expressed (Figure 7A). The overlap between genes differentially expressed in CD103 positive cells from CT and CD was low (Figure 7B), suggesting major changes in the transcriptomic landscape in CD103 positive CD8 T cells in CD.

We next focused on the differentially expressed genes implicated in immunological functions of CD103 positive and negative cells. As expected, the expression of ITGAE, encoding the integrin αE and its associated signaling molecule, integrin-linked kinase (ILK), were upregulated in CD103 positive CD8 T cells (Figures 7C,D). In accordance with our flow cytometry data, KLRC1 encoding NKG2A was upregulated the CD103 positive subset (Figure 2 and Figures 7C,D). KLRC2 encoding NKG2C, from the same family of receptors was also increased in CD103 positive cells (Figures 7C,D). Interestingly, TCR signaling and T cells activation markers such as CD3E, nuclear factor of activated T cells (NFAT5) and IL2RA were also upregulated in CD103 positive compared to negative cells (Figures 7C,D), which could explain the increase responsiveness of CD103 positive cells to TCR triggering *ex vivo* (Figure 5).

Genes previously described (8) as expressed in intestinal Trm cells such as CCR9 and AHR were more expressed in CD103 positive T compared to negative cells (Figures 7C,D). CD103 positive T cells displayed increase expression of transcript encoding regulatory cytokine IL10 and its specific receptor IL10RB, but also pro-inflammatory cytokine such as IL15 and TNFα as well as immune cell recruiting factors such as XCL1 and CXCL16 (Figures 7C,D). These results suggest that CD103 positive cells actively participate to the general immune response in the intestinal mucosa.

### CD103 CD8 T Cells From CD Patient Display Increase Th17 Related Gene and GZM K Expression

**Figure 8:**
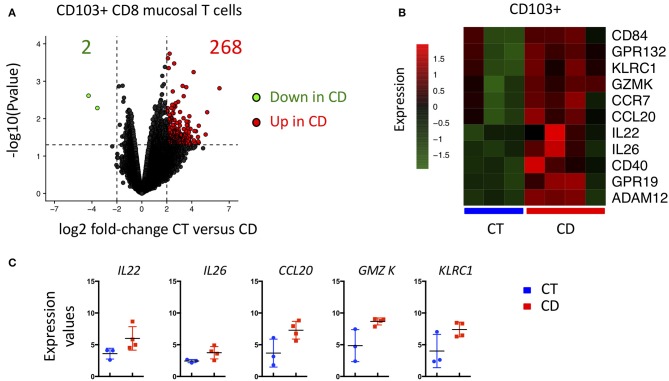
CD103+ CD8 Trm cells from CD patient display major transcriptional change compared to CD103+ from CT. **(A)** Volcanoplot showing differential expression (fold change >2 and *P* < 0.05) in CD103+ between control (CT; *n* = 3) and CD patients (CD; *n* = 4). **(B)** Heatmap of genes differentially expressed (*P* < 0.05) between CD103+ mucosal T cells from CT and CD patients. **(C)** Quantification and distribution of expression value of genes of interest (dots and bars represent 3 and 4 independent experiments with mean and SEM).

Given the major changes in the transcriptomic landscape of CD103 positive CD8 T cells in CD, we compared them to cells isolated from control ileum (CT). In accordance with our precedent analyses, a large number of genes were significantly upregulated in CD103 CD8 T cells from CD patients compared to CT (Figure 8A). KLCR1 encoding NKG2A and GZMK were also increased in CD103 positive CD8 T cells of CD patients compared to CT (Figures 8B,C). CD103 Trm cells from CD expressed more Th17 related cytokines such as IL22, IL26, and CCL20 (Figures 8B,C), cytokines and chemokines involved in intestinal tissue homeostasis and recruitment of immune cells. Taken together, these results show that CD103 CD8 T cells are strongly modified in CD patients and suggest that they could be important players in the pathological immune response arising in these patients.

## Discussion

Here we show that CD8 T cells can differentially express adhesion, activatory and inhibitory molecules. Expressions of CD103 and KLRG1 are mutually exclusive and hence could define two major subsets of CD8 Trm cells in the mucosa. Indeed, CD103 CD8 T cells can co-express NKG2A and are more responsive to TCR triggering. On the other hand, KLRG1 CD8 T cells express high intracellular levels of GZM B and Ki67, showing their cytotoxic and proliferative potential, and are increased in the intestinal mucosa of CD patients. Interestingly, CD103 CD8 T cells originate from KLRG1 negative precursors after TCR triggering and TGFβ stimulation. Transcriptomic analyses show major modification of CD103 CD8 T cells from the mucosa of CD patients compared to CT, with increase expression of immune modulating molecules.

Our study suggests that CD8 Trm cells have a major impact in intestinal homeostasis and could participate to the physiopathology of CD. However, the exact role of CD8 Trm cells in the mucosa and the impact of different subsets remain elusive. We recently published that persistent clonal expansions in the intestinal mucosa, mostly in the CD8 T cell compartment, are associated with and predictive of disease recurrence in patients undergoing surgery (26). It would be of great interest to study the clonality of the different CD8 Trm subsets in the intestine and to determine if the same CD8 clone differentiates into both CD103 and KLRG1 CD8 T cells.

Indeed, recent studies in mice have shown that the same clones are found in CD8 Tcm in the lymph nodes and CD8 Trm in the tissues indicating that the same naive T cells can differentiate into several subsets of CD8 memory cells. Mouse models using adoptive transfer and fate mapping show that KLRG1 positive memory CD8 T cell can differentiate into circulating Tem, Tcm and distinct Trm subsets in the mucosa expressing KLRG1 or CD103 (18, 27). We show that CD103 expressing human CD8 T cells arise from responsive KLRG1 negative precursors following TGFβ and TCR stimulation, while KLRG1 positive cells may be recruited and accumulate in the intestinal mucosa in inflammatory conditions. These results suggest that CD8 Trm cells could encompass different subset of cells with heterogeneity in their functions and locations, which could have a role in the homeostasis of the intestine.

KLRG1 CD8 T cells are mostly present in the blood and in low proportions in healthy intestinal mucosa, which could be explained by the poor survival of these cells in tissues (19, 27). We observed more KLRG1 positive CD8 T cells in CD patient's mucosa compared to CT, which could result from the survival signals provided by the intestinal proinflammatory cytokine in CD, such as IL-15, and/or their response to a cognate antigen. This phenomenon has been observed in infection mouse models where TGFβ-independent CD103-negative CD8 Trm cells have been described in response to a specific antigen (20).

The distinction between human KLRG1 and CD103 CD8 Trm cell functions is still unclear, especially in pathologic background like CD. We find that KLRG1 CD8 T cells from blood and mucosa are positive for intracellular GZM B, while CD103 CD8 T cells expressed low levels of this cytotoxic mediator. This is in accordance with previous studies on CD103 CD8 Trm cells from human lung (7, 28) and brain (29). On the other hand, CD103 CD8 T cells are more responsive to TCR stimulation, which could be explained by increase expression of TCR signaling molecules such as CD3E and increase response through IL2RA. Moreover, CD103 CD8 T cells also expressed more pro-inflammatory cytokines such as IL-15 and TNFα. These results suggest that both KLRG1 and CD103 CD8 T cells could participate to local inflammation through granzyme and cytokine-dependent mechanisms, respectively.

CD103 CD8 T cells from CD patients express higher levels of IL22, IL26 and CCL20, three mediators associated with the Th17 pathway (30–34), compared to cells isolated from control mucosa. IL-26 have direct antimicrobial activity by killing extracellular bacteria via membrane-pore formation and promote immune sensing of bacteria by innate immune receptor (35). IL-22 is known to induce antimicrobial peptides production by Paneth cells and proliferation of IEC, which prevent tissue damage and promote tissue-healing (36). CCL20 chemokine is known to attract CCR6 expressing cells such as CD4 T cells, T regulatory and B cells. These mediators have beneficial action on the mucosa by promoting antimicrobial activity, immune sensing and tissue repair but also promote inflammation through cytokine and chemokine productions (such as TNFα, IL-8 and CCL20) (32, 36) and recruitment of new effector cells including KLRG1 CD8 T cells that express CCR6.

Our results suggest that CD8 T cells could be major partners in the regulation of the immune system in the intestine in both health and disease, through effector or regulatory functions in both health and disease (37, 38). The study of tissue resident cells in human is a challenge as the availability of adequate controls is missing. Advances in molecular and cytometry techniques allow for the study of cell population isolated from tissues in high dimensions. Single-cell analyses of immune cells from IBD patients recently determined a transcriptional signature associated with disease activity and response to treatments (39). Combining single-cell and TCR repertoire analyses would define the capacity of one clone to acquire different phenotype in the intestinal mucosa. However, this type of approach still necessitate fresh samples, which is difficult to apply to the study of large cohort of patients that are necessary to mitigate interindividual heterogeneity.

Hence, CD8 Trm cells are local sensors poised for immediate pathogen interception and clearance through diverse functions including direct cytotoxicity, cytokine production increasing expression of innate immune gene and chemokine release to recruit other effectors cell. Our finding suggest that CD103 CD8 Trm cells in CD induce a tissue-wide alert by increasing innate immune responses and recruiting other effector cells such as KLRG1 CD8 Trm cells.

## Methods

### CD Patients and Controls

Surgical samples were obtained from ileal bowel resection of CD and right colon cancer patients at the Saint-Louis Hospital. In CD patients, cells were isolated from the inflamed and non-inflammed areas of resected specimens (referred as Inf and Non-inf, respectively). The study was approved by AFFSAPS (IDRCB: 2009-A00205-52) and the French Ethic Committee (CPP 2009/17) and declared to ClinicalTrials.gov (NCT03458195). In the control group, cells were isolated on normal ileal tissue located at least 10 cm away from tumor (referred as CT) as part of another study (CPP Ile de France IV: 2016/45). Blood of healthy donor were provided by EFS (Etablissement Français du Sang).

### Cells Sorting and Flow Cytometry

Cell isolation from surgical specimens was performed as previously described (37). Magnetic bead-conjugated antibodies to CD5 were used to isolate T cells from PBL from healthy donor for *in vitro* test using an autoMACSPro Separator (Miltenyi Biotec, Paris, France). To isolate CD45RO- and CD45RO+ T-cells from PBL or mucosal CD103+ and CD103- CD8 γδ- T cells, cells were sorted with Aria II or III using antibodies against CD4 Pecy7, CD5 AF700, CD8 BV605 (all BD Bioscience), CD45RO Pecy5, TCRγδ FITC, and CD103 APC-vio770, KLRG1 PE antibodies (all from Myltenyi Biotech).

CD and control patient phenotypes were performed using CD4 BV711, CD8 BV605, CD3 AF700, CD45RO PECy5, GZM B APC (all BD Bioscience), CCR6 PE, CD103 APC-vio770, KLRG1 PEvio770 and NKG2A FICT antibodies (all from Myltenyi Biotech). 4,6-diamidino-2-phenylindole (DAPI) (Sigma) was used to detect live cells. Data were acquired on an Attune NxT Flow Cytometer (ThermoFischer) using attune software. All data analyses were performed with flowJo 10.4.2 and cytobank (www.cytobank.com) for t-SNE visualization.

### *In vitro* T Cells Culture Conditions

Magnetic sorted CD5+ and Aria sorted CD5+ CD8+ CD45RO+/- T cells were incubated with optimized dose of IL-15 (10 ng/ml) and TGFb (20 ng/ml) during 7 days with or without CD3/CD28 stimulation using commercially available coated beads (all from Myltenyi Biotech).

Mucosal T cells from CD patients were stimulated by CD3 using coated beads. Briefly Anti-Biotin MACS iBead Particles (130-092-357, Myltenyi Biotech) were coated with IgG1, CD3 biotin-labeled antibodies following manufacturer instruction. (Antibodies concentrations are the same in all conditions). Then, T cells were cultured and stimulated by the beads in cytokine-free medium during 24 h before analysis by flow cytometry.

### Statistical Analysis and Data Visualization

Statistical analysis and data visualization was performed with graphpad prism version (grapPad Software, Inc., La Jolla) or R software. Paired or unpaired *T*-test or ANOVA was used to test significance of the difference between group of patient and *in vitro* experiment. Statistical significance was defined as *p* < 0.05. *P*-value are represented as following: ^*^P < 0.05, ^**^P < 0.001, ^***^P < 0.0001, ^****^P < 0.00001.

### RNA Extraction and Microarray

Total RNA was extracted and purified using RNeasy micro kit (Qiagen), according to the manufacturer's instructions. The quantity and purity of total isolated RNA were determined by measuring the absorbance at 260 nm and 280 nm using a NanoDrop 2000 Spectrophotometer (Thermo Fisher Scientific). Samples were then tested for quality with the Caliper LabChip GX High-Throughput Bioanalyzer (Life Sciences).

RNA quantification and quality control was performed using the HT RNA Pico sensitivity LabChip Kit and the Caliper LabChip Microfluidics System (Perkin Elmer). Four nanogrammes of Total RNA was amplified, labeled, and fragmented using GeneChip WT Pico (ThermoFisher Scientific). Each sample was hybridized onto Human Clariom D (ThermoFisher Scientific), washed, and stained with the Affymetrix® Fluidics Station 450. Array Scanning was performed with the Affymetrix® GeneChip Scanner 3000 7G using the Command Console software (ThermoFisher Scientific). and then analyzed using the Affymetrix® rma-sketch routine.

The microarray raw data were preprocessed to obtain log2 expression value and normalized using robust multi-array average (RMA) method as implemented in the affy R package. Statistical analysis of differentially expressed genes between groups was performed using the linear models for microarray (LIMMA) R package. Hierarchical clustering was obtained using Euclidean distance and average linkage to visualize transcriptome-associated clusters. All graphs and statistics were performed using R software (version 3.3.0).

### CD103 and Granzyme B Immunostaining

Three micrometers thickness ileal resection margin tissue sections fixed on polarized slides were first deparaffinised and rehydrated with successive baths of Xylenes isomeric mixtures and Ethanol at different concentrations. Antigen retrieval was performed using Citrate buffer (pH 6,0; Sigma Aldrich®) heated in a microwave. For immunohistochemistry, endogenous peroxidase activity was inhibited using hydrogen peroxide. After blocking unspecific bonds with blocking buffer (prepared with a 5% species' serum of the secondary antibody), staining with primary antibodies was performed. For CD103 and Granzyme B immunohistochemistry, primary was detected with an iVIEW DAB Detection Kit (Ventana ®) using an automated system. CD103 and Granzyme B stainings were performed using primary antibody from abcam (ab#129202, Dilution 1/500, Rabbit mAb) and DAKO (ab#M7235, Dilution 1/50, Mouse mAb). CD103 and Granzyme B staining intensities of the whole mucosa were evaluated by image processing for quantification analysis. Qupath ® software was used for all analyses. Scanned proximal resection margins were uploaded in the software. Mucosa was delimited using the polygon tool. Positive cell detection was then performed automatically. Positive cell rates were then extracted and comparative statistical analyses were performed. The epithelium and the lamina propria were then distinguished using the detection classifier tool by machine learning. Positive cell rates for both the epithelium and the lamina propria were then extracted and comparative statistical analyses were performed.

## Data Availability Statement

The raw data supporting the conclusions of this article will be made available by the authors, without undue reservation, to any qualified researcher.

## Ethics Statement

The studies involving human participants were reviewed and approved by Comité de protection des personnes (CPP) Ile-de-France IV. The patients/participants provided their written informed consent to participate in this study.

## Author Contributions

HB and LL designed the experiments and interpreted the data. HB helped by JB and TC performed *in vitro* experiments. VC, CG, and BG handled and processed patient samples. HB and MN performed RNA extraction, microarray experiments and bioinformatics data analysis. NH performed histological experiments and quantification. HB, MA, and LL wrote the manuscript. All authors read and approved the final manuscript.

## Conflict of Interest

The authors declare that the research was conducted in the absence of any commercial or financial relationships that could be construed as a potential conflict of interest.

## References

[1] Marzo AL Klonowski KD Le Bon A Borrow P Tough DF Lefrançois L. Initial T cell frequency dictates memory CD^8+^ T cell lineage commitment. Nat Immunol. (2005) 6:793–9. 6:793–9. 10.1038/ni1227 16025119 PMC2849311

[2] Obar J Lefrançois L. Memory CD^8+^ T cell differentiation. Ann Ny Acad Sci. (2010) 1183:251–66. 10.1111/j.1749-6632.2009.05126.x 20146720 PMC2836783

[3] Cui W Kaech S. Generation of effector CD^8+^ T cells and their conversion to memory T cells. Immunol Rev. (2010) 236:151–66. 236:151–66. 10.1111/j.1600-065X.2010.00926.x 20636815 PMC4380273

[4] Schenkel JM Masopust D. Tissue-Resident memory T Cells. Immunity. (2014) 41:886–97. 41:886–97. 10.1016/j.immuni.2014.12.007 25526304 PMC4276131

[5] Kumar BV Ma W Miron M Granot T Guyer RS Carpenter DJ. Human tissue-resident memory t cells are defined by core transcriptional and functional signatures in lymphoid and mucosal sites. Cell Rep. (2017) 20:2921–34. 10.1016/j.celrep.2017.08.078 28930685 PMC5646692

[6] Hombrink P Helbig C Backer RA Piet B Oja AE Stark R. Programs for the persistence, vigilance and control of human CD8(+) lung-resident memory T cells. Nat Immunol. (2016) 17:1467–78. 17:1467–78. 10.1038/ni.3589 27776108

[7] Mackay LK Kallies A. Transcriptional regulation of tissue-resident lymphocytes. Trends Immunol. (2017) 38:94–103. 10.1016/j.it.2016.11.004 27939451

[8] Mackay LK Rahimpour A Ma JZ Collins N Stock AT Hafon M-L. The developmental pathway for CD103(+)CD8(+) tissue-resident memory T cells of skin. Nat. Immunol. (2013) 14:1294–301. 14:1294–301. 10.1038/ni.2744 24162776

[9] Mueller S Mackay L. Tissue-resident memory T cells: local specialists in immune defence. Nat Rev Immunol. (2015) 16:79–89. 10.1038/nri.2015.3 26688350

[10] Agace W. T-cell recruitment to the intestinal mucosa. Trends Immunol. (2008) 29:514–22. 29:514–22. 10.1016/j.it.2008.08.003 18838302

[11] Mackay LK Wynne-Jones E Freestone D Pellicci DG Mielke LA Newman DM. T-box transcription factors combine with the cytokines TGF-β and IL-15 to control tissue-resident memory T cell fate. Immunity. (2015) 43:1101–11. 43:1101–11. 10.1016/j.immuni.2015.11.008 26682984

[12] Mucida D Park Y Cheroutre H. From the diet to the nucleus: vitamin A and TGF-β join efforts at the mucosal interface of the intestine. Semin Immunol. (2009) 21:14–21. 21:14–21. 10.1016/j.smim.2008.08.001 18809338 PMC2643336

[13] Reis BS Rogoz A Costa-Pinto FA Taniuchi I Mucida D. Mutual expression of the transcription factors Runx3 and ThPOK regulates intestinal CD4+ T cell immunity. Nat. Immunol. (2013) 14:271–80. 14:271–80. 10.1038/ni.2518 23334789 PMC3804366

[14] Sheridan BS Pham QM Lee YT Cauley LS Puddington L Lefrançois L. Oral infection drives a distinct population of intestinal resident memory CD8(+) T cells with enhanced protective function. Immunity. (2014) 40:747–57. 10.1016/j.immuni.2014.03.007 24792910 PMC4045016

[15] Cauley LS Lefrançois L. Guarding the perimeter: protection of the mucosa by tissue-resident memory T cells. Mucosal Immunol. (2013) 6:14–23. 10.1038/mi.2012.96 23131785 PMC4034055

[16] Mackay LK Braun A Macleod BL Collins N Tebartz C Bedoui S. Cutting edge: CD69 interference with sphingosine-1-phosphate receptor function regulates peripheral T cell retention. J Immunol Mar. (2015) 194:2059–63. 10.4049/jimmunol.1402256 25624457

[17] Bergsbaken T Bevan MJ. Proinflammatory microenvironments within the intestine regulate the differentiation of tissue-resident CD8(+) T cells responding to infection. Nat Immunol. (2015) 16:406–14. 10.1038/ni.3108 25706747 PMC4368475

[18] Rosshart S Hofmann M Schweier O Pfaff A Yoshimoto K Takeuchi T. Interaction of KLRG1 with E-cadherin: new functional and structural insights. Eur J Immunol. (2008) 38:3354–64. 38:3354–64. 10.1002/eji.200838690 19009530

[19] Gründemann C Bauer M Schweier O von Oppen N Lässing U Saudan P. Cutting edge: identification of E-cadherin as a ligand for the murine killer cell lectin-like receptor G1. J Immunol. (2006) 176:1311–5. 10.4049/jimmunol.176.3.1311 16424155

[20] Gaide O Emerson RO Jiang X Gulati N Nizza S Desmarais C. Common clonal origin of central and resident memory T cells following skin immunization. Nat Med. (2015) 21:647–53. 21:647–53. 10.1038/nm.3860 25962122 PMC4632197

[21] Bartolomé-Casado R Landsverk OJB Chauhan SK Richter L Phung D Greiff V. Resident memory CD8 T cells persist for years in human small intestine. J Exp Med Oct. (2019) 216:2412–26. 216:2412–26. 10.1084/jem.20190414 31337737 PMC6781004

[22] Park CO Kupper TS. The emerging role of resident memory T cells in protective immunity and inflammatory disease. Nat Med. (2015) 21:688–97. 10.1038/nm.3883 26121195 PMC4640452

[23] Clark RA. Resident memory T cells in human health and disease. Sci Transl Med. (2015) 7:269rv1. 10.1126/scitranslmed.3010641 25568072 PMC4425129

[24] Cheuk S Schlums H Gallais Sérézal I Martini E Chiang SC Marquardt N. CD49a expression defines tissue-Resident CD8+ T cells poised for cytotoxic function in human skin. Immunity. (2017) 46:287–300. 10.1016/j.immuni.2017.01.009 28214226 PMC5337619

[25] Meresse B Malamut G Cerf-Bensussan N. Celiac disease: an immunological jigsaw. Immunity. (2012) 36:907–19. 10.1016/j.immuni.2012.06.006 22749351

[26] Allez M Auzolle C Ngollo M Bottois H Chardiny V Corraliza AM. T cell clonal expansions in ileal crohn's disease are associated with smoking behaviour and postoperative recurrence. Gut. (2019) 68:1961–70. 10.1136/gutjnl-2018-317878 30792246

[27] Herndler-Brandstetter D Ishigame H Shinnakasu R Plajer V Stecher C Zhao J. KLRG1+ effector CD8+ T Cells Lose KLRG1, differentiate into all memory T cell lineages, and convey enhanced protective immunity. Immunity. (2018) 48:716–29.e8. 48:716–29.e8. 10.1016/j.immuni.2018.03.015 29625895 PMC6465538

[28] Piet B de Bree GJ Smids-Dierdorp BS van der Loos CM Remmerswaal EB von der Thüsen JH CD8+ T cells with an intraepithelial phenotype upregulate cytotoxic function upon influenza infection in human lung. J Clin Invest. (2011) 121:2254–63. 10.1172/JCI44675 21537083 PMC3104744

[29] Smolders J Heutinck KM Fransen NL Remmerswaal EBM Hombrink P Iten Berge JM. Tissue-resident memory T cells populate the human brain. Nat Commun. (2018) 9:4593. 9:4593. 10.1038/s41467-018-07053-9 30389931 PMC6214977

[30] Fujii M Nishida A Imaeda H Ohno M Nishino K Sakai S. Expression of interleukin-26 is upregulated in inflammatory bowel disease. World J Gastroenterol. (2017) 23:5519–29. 23:5519–29. 10.3748/wjg.v23.i30.5519 28852311 PMC5558115

[31] Dambacher J Beigel F Zitzmann K De Toni EN Göke B Diepolder HM. The role of the novel Th17 cytokine IL-26 in intestinal inflammation. Gut. (2009) 58:1207–17. 58:1207–17. 10.1136/gut.2007.130112 18483078

[32] Bamias G Cominelli F. Cytokines and intestinal inflammation. Curr Opin Gastroenterol. (2016) 32:437–42. 10.1097/MOG.0000000000000315 27673380

[33] Li LJ Gong C Zhao MH Feng BS. Role of interleukin-22 in inflammatory bowel disease. World J Gastroenterol. (2014) 20:18177–88. 20:18177–88. 10.3748/wjg.v20.i48.18177 25561785 PMC4277955

[34] Brand S Beigel F Olszak T Zitzmann K Eichhorst ST Otte JM. IL-22 is increased in active Crohn's disease and promotes proinflammatory gene expression and intestinal epithelial cell migration. Am J Physiol Gastrointest Liver Physiol. (2006) 290:G827–38. 290:G827–38. 10.1152/ajpgi.00513.2005 16537974

[35] Meller S Di Domizio J Voo KS Friedrich HC Chamilos G Ganguly D. T(H)17 cells promote microbial killing and innate immune sensing of DNA via interleukin 26. Nat Immunol. (2015) 16:970–9. 16:970–9. 10.1038/ni.3211 26168081 PMC4776746

[36] Kaser A Ludwiczek O Holzmann S Moschen AR Weiss G Enrich B. Increased expression of CCL20 in human inflammatory bowel disease. J Clin Immunol. (2004) 24:74–85. 24:74–85. 10.1023/B:JOCI.0000018066.46279.6b 14997037

[37] Corgnac S Boutet M Kfoury M Naltet C Mami-Chouaib F. The emerging role of CD8+ tissue resident memory T (TRM) cells in antitumor immunity: a unique functional contribution of the CD103 integrin. Front Immunol. (2018) 9:1904. 10.3389/fimmu.2018.01904 30158938 PMC6104123

[38] Allez M Brimnes J Dotan I Mayer L. Expansion of CD8+ T cells with regulatory function after interaction with intestinal epithelial cells. Gastroenterology. (2002) 123:15161526. 123:15161526. 10.1053/gast.2002.36588 12404227

[39] Martin JC Chang C Boschetti G Ungaro R Giri M Grout JA. Single-Cell analysis of crohn's disease lesions identifies a pathogenic cellular module associated with resistance to Anti-TNF therapy. Cell Sep. (2019) 178:1493–508.e20. 10.1016/j.cell.2019.08.008 31474370 PMC7060942

