# Supplementary material for: KLRG1 and CD103 Expressions Define Distinct Intestinal Tissue-Resident Memory CD8 T Cell Subsets Modulated in Crohn's Disease
Source: Front Immunol. 2020 May 12;11:896. doi: 10.3389/fimmu.2020.00896 (PMC7235448; doi:10.3389/fimmu.2020.00896)
Supplement: Supplementary file 1 [file Image_1.pdf]

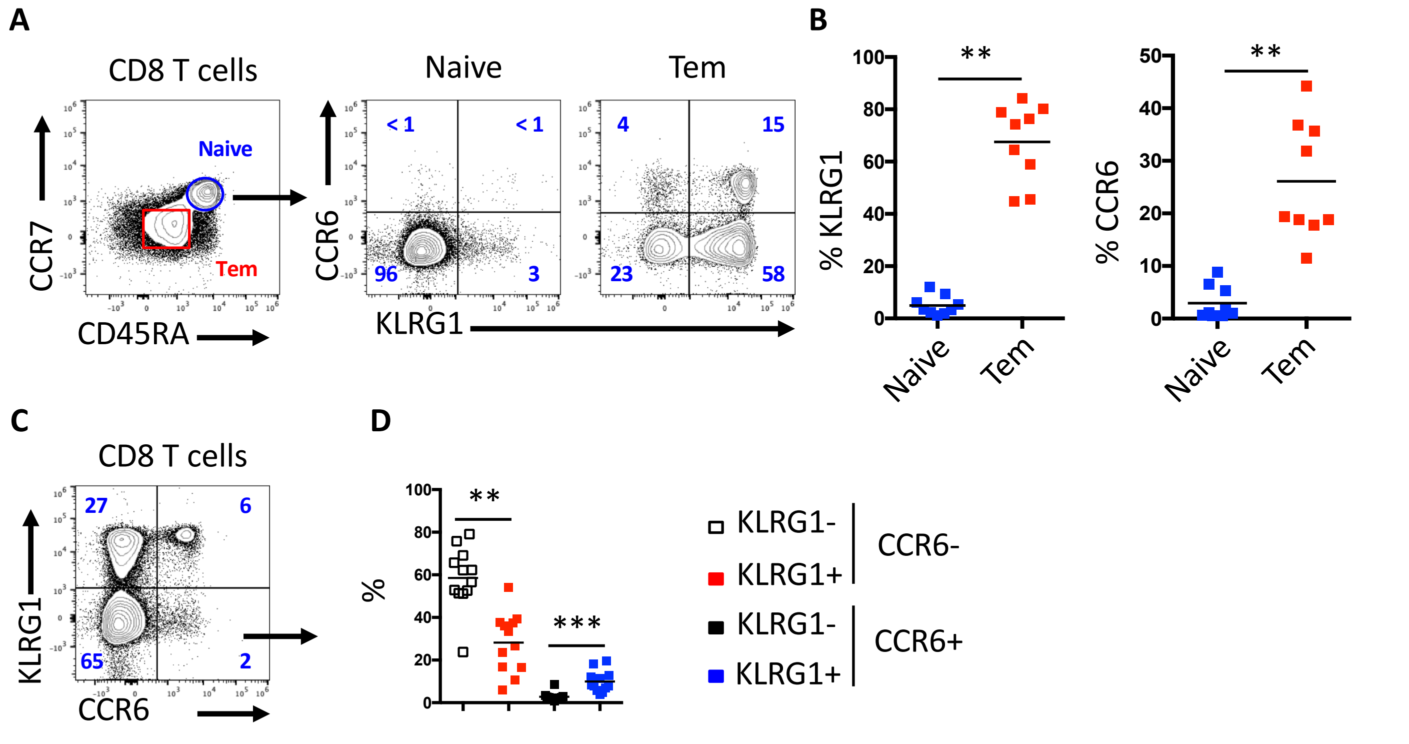

Supplementary Figure 1: Effector memory CD8 T expression of CCR6 (A) Expression and quantification (B) of CCR6 and KLRG1 by naive and effector memory CD8 T cells (n=10; bars represent mean, paired t-test : ** p<0,001). (C) Coexpression and (D) quantification of CCR6-KLRG1- (white), CCR6-KLRG1+ (red), CCR6+KLRG1- (black) and CCR6+KLRG1+ (blue) cells in total CD8 T cells (n=10; bars represent mean, paired t-test : ** p<0.001; *** p<0.001).

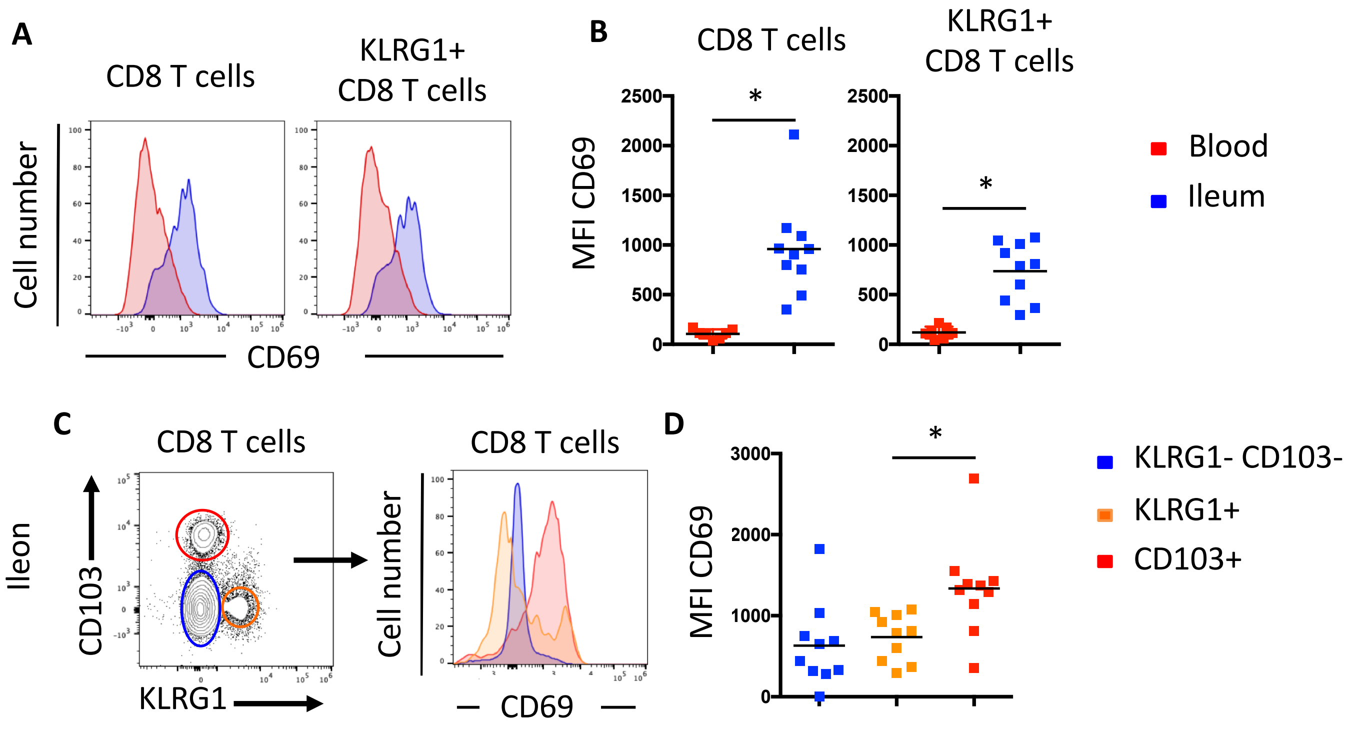

Supplementary Figure 2: Mucosal KLRG1 positive T cells express Tissue resident memory markers CD69 (A) Expression of CD69 on KLRG1 and (B) quantification of positive CD8 T cells from blood and mucosa of CD patients (n=10; bars represent mean, paired t-test : * $p<0.05$). (C) Expression and (D) quantification of CD69 marker by CD103, KLRG1 and double negative mucosal CD8 T cell subset (n=10; bars represent mean, paired t-test : * $p<0.05$).

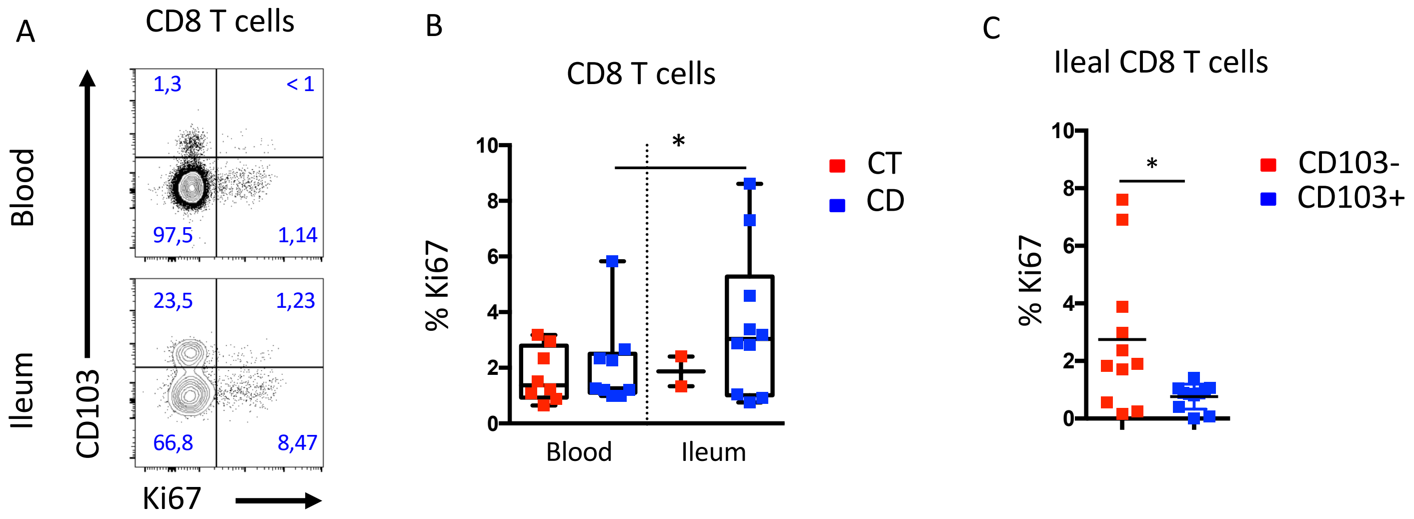

Supplementary Figure 3: Differential expression of Ki67 and GZM B between CD8 Trm cells and blood T cells. (A) Expression of CD103, Ki67 and GZMB on CD8 T cells in blood and ileal mucosa. (B) Quantification of GZMB and Ki67 on total CD8 T cells in blood of CT and blood and ileal mucosa of CD patients (n=10 or n=2 for control ileum; bars represent mean, paired t-test : * p<0.05). (C) Quantification of Ki67 expression on CD103+ and CD103- T cell subsets (n=10; bars represent means; paired t-test: * p<0.05).
